# Structure of the herpes simplex virus portal-vertex

**DOI:** 10.1371/journal.pbio.2006191

**Published:** 2018-06-20

**Authors:** Marion McElwee, Swetha Vijayakrishnan, Frazer Rixon, David Bhella

**Affiliations:** Medical Research Council, University of Glasgow Centre for Virus Research, Glasgow, United Kingdom; University of Wisconsin-Madison, United States of America

## Abstract

Herpesviruses include many important human pathogens such as herpes simplex virus, cytomegalovirus, varicella-zoster virus, and the oncogenic Epstein–Barr virus and Kaposi sarcoma–associated herpesvirus. Herpes virions contain a large icosahedral capsid that has a portal at a unique 5-fold vertex, similar to that seen in the tailed bacteriophages. The portal is a molecular motor through which the viral genome enters the capsid during virion morphogenesis. The genome also exits the capsid through the portal-vertex when it is injected through the nuclear pore into the nucleus of a new host cell to initiate infection. Structural investigations of the herpesvirus portal-vertex have proven challenging, owing to the small size of the tail-like portal-vertex–associated tegument (PVAT) and the presence of the tegument layer that lays between the nucleocapsid and the viral envelope, obscuring the view of the portal-vertex. Here, we show the structure of the herpes simplex virus portal-vertex at subnanometer resolution, solved by electron cryomicroscopy (cryoEM) and single-particle 3D reconstruction. This led to a number of new discoveries, including the presence of two previously unknown portal-associated structures that occupy the sites normally taken by the penton and the Ta triplex. Our data revealed that the PVAT is composed of 10 copies of the C-terminal domain of pUL25, which are uniquely arranged as two tiers of star-shaped density. Our 3D reconstruction of the portal-vertex also shows that one end of the viral genome extends outside the portal in the manner described for some bacteriophages but not previously seen in any eukaryote viruses. Finally, we show that the viral genome is consistently packed in a highly ordered left-handed spool to form concentric shells of DNA. Our data provide new insights into the structure of a molecular machine critical to the biology of an important class of human pathogens.

## Author summary

The herpesvirus family includes many important human pathogens such as herpes simplex viruses that cause cold-sores and human cytomegalovirus, a major cause of congenital abnormalities. Several herpes viruses are known to cause cancer. Herpes viruses assemble enveloped virus particles (virions) that incorporate a large DNA-containing icosahedral capsid. Virion assembly commences in the nucleus of an infected cell, where the viral genome is pumped into preassembled capsids by a portal motor that is located at a unique 5-fold symmetry axis: the portal-vertex. We have used cryogenic electron microscopy and 3D image reconstruction to solve the structure of the portal-vertex of herpes simplex virus type 1 at 8 Å resolution. Our structure reveals the presence of several previously unknown features including a novel pentameric assembly that exhibits a coiled-coil motif comprising two α-helices. This substitutes the penton, a pentamer of the major capsid protein that is seen at other 5-fold symmetry axes (penton-vertices). Our data allowed us to postulate an identity for the small tail-like structure previously identified at the portal-vertex and termed the portal-vertex–associated tegument (PVAT). Finally, our 3D reconstruction reveals that the viral DNA is packaged within the capsid as a left-handed spool that is arranged in concentric shells. Our data present a structural view of a molecular machine that plays a critical role in the replication cycle of an important family of human pathogens.

## Introduction

Herpes Simplex Virus 1 and 2 (HSV-1 and HSV-2) are important human pathogens. It is estimated that approximately 90% of the world’s population are infected with one or both viruses [1]. HSV-1 is the primary cause of cold sores and HSV-2 of genital herpes. These conditions are both highly contagious, and HSV-2 is amongst the most common sexually transmitted infections. Infection with HSV is lifelong, owing to the ability of herpesviruses to enter a latent state with periodic reactivations [2]. HSV can also cause more serious conditions including keratitis, which may lead to loss of sight [3], and a potentially fatal encephalitis [4]. The herpesvirus family includes many other important human pathogens, such as varicella-zoster virus, the cause of chicken pox and shingles; cytomegalovirus, a notable cause of congenital abnormalities; Kaposi sarcoma–associated herpesvirus, which causes cancer in immune-compromised individuals; and Epstein–Barr virus, the cause of infectious mononucleosis that has also been linked to several cancers.

Herpesviruses are large double-stranded DNA viruses, having genomes up to 240 kbp. The viral DNA is packaged in a complex T = 16 icosahedral capsid that is 1,250 Å in diameter [5,6]. The DNA-containing capsid, or nucleocapsid, is embedded in a proteinaceous layer known as the tegument that is in turn surrounded by a host-derived lipid envelope. The viral envelope is studded with glycoproteins that mediate viral attachment and entry. HSV virions enter host cells by fusing their envelopes with the host cell plasma membrane, allowing the nucleocapsid and tegument to enter the cytoplasm [7]. The nucleocapsid traffics along microtubules to the microtubule-organising centre, and from there, to the nucleus [8]. The nucleocapsid then docks to a nuclear pore complex, through which it injects its genome into the nucleus [9,10]. DNA egress from the capsid is through a unique portal-vertex, located at an icosahedral 5-fold symmetry axis.

The portal-vertex is also the means by which the viral DNA is packaged into capsids within the nucleus [11]. Virion morphogenesis commences in the nucleus with the formation of the procapsid; an icosahedrally symmetrical spherical shell assembly of capsomeres that are hexamers (hexons) and pentamers (pentons) of the major capsid protein pUL19 (VP5) [12]. Heterotrimers of pUL38 (VP19C) and pUL18 (VP23)—termed triplexes—along with the scaffold protein pUL26, direct procapsid assembly, which nucleates around the dodecameric portal formed by pUL6 [13]. Procapsid maturation—angularisation and expulsion of the scaffold protein—occurs as the viral genome is pumped into the shell [14]. Replication of the viral genome results in formation of a concatemer, from which unit-length genomes are packaged into procapsids by the portal (pUL6) and terminase complex consisting of pUL33, pUL28, and pUL15 [15,16]. It has been noted that herpesviruses are structurally and biologically similar to DNA-containing tailed bacteriophages, and it has been suggested that these two viral groups share a common ancestry. This is based on the observation of fold conservation in the major capsid protein [17] and their similar capsid assembly and DNA-packaging strategies. Sequence analysis also indicates that pUL15 is a homolog of the phage terminase large subunit. By analogy, pUL15 is predicted to have ATPase activity, powering the translocation of the genome into the capsid through the portal, and endonuclease activity cleaving the DNA when a cleavage signal is detected [18]. pUL28 is known to bind viral DNA and is equivalent to the small terminase subunit of bacteriophages [19].

The capsid-associated tegument complex (CATC—previously termed CCSC and CVSC), is composed of pUL17, pUL25, and pUL36 and binds to the triplexes and hexons about the icosahedral 5-fold vertices of mature capsids within the nucleus [5,20–25]. Notably, it has been observed that pUL25 is essential for retention of DNA within the nucleocapsid [26,27]. Moreover, pUL25 has also been shown to be important for genome release [28].

The mature nucleocapsid leaves the nucleus by budding through the nuclear membrane via an envelopment/de-envelopment step [29]. Cytoplasmic capsids acquire further tegument proteins in the cytoplasm and are enveloped by budding into plasma membrane–derived lipid vesicles, from which they are released by exocytosis at the cell surface [30].

The structure of the HSV particle has been the subject of investigation for over 30 years [31], using cryoEM and icosahedral 3D reconstruction to determine the high-resolution features of the nucleocapsid [5,25,32,33] and lower-resolution tomography to investigate the nature of asymmetric features such as the portal-vertex and viral envelope [34,35]. Attempts to resolve the structure of the portal-vertex have been largely unsuccessful, however. This is because the herpesvirus portal-vertex is similar in size and mass to the penton-vertex, unlike bacteriophages, in which the portal-vertex is marked by the presence of a substantial tail assembly. Moreover, in herpes virions, the subtle differences between the portal-vertex and penton-vertices are obscured by the tegument layer. Finally, the high symmetry of the viral capsid dominates attempts to align particle images for asymmetric reconstruction.

Here, we show the structure of the portal-vertex of HSV-1 at 8 angstroms resolution, revealed by focussed-classification [36,37] and 3D reconstruction of cryoEM images of purified virions. These data reveal that the usual pUL19 penton is replaced by a unique 5-fold symmetrical assembly. This feature displays five well-defined coiled-coil motifs, each made up of two α-helices, arranged perpendicular to the capsid surface about the 5-fold symmetry axis. It appears to be anchored to the virion by interactions with triplex-like structures that occupy the position normally taken up by peripentonal Ta triplexes, immediately about the 5-fold vertex. The CATC assembly is still present and, similarly to penton associated CATC, is bound to the Tc triplexes, forming a bridge across the periportal triplex-like structures towards the 5-fold axis. We interpret our data as showing that the pUL25 C-terminal domains are positioned differently to those seen at penton-vertices, giving rise to a small tail-like assembly that crowns the unique 5-fold vertex. Strong density was seen to extend through the portal-vertex structures that we interpret as DNA. This suggests that the trailing end of the packaged genome remains engaged in the portal-vertex, ready for release through the nuclear pore. The portal itself is not well resolved owing to a mismatch between the C5 symmetry imposed in calculating our reconstruction and the C12 symmetry of that feature. Finally, our reconstruction also reveals the arrangement of packaged DNA within the virion, which is clearly resolved as a left-handed spool arranged in concentric layers.

We provide the highest-resolution view to date of a critical component of the herpesvirus virion. The portal-vertex is a molecular machine responsible for both packaging and release of the viral genome, in one of the most important groups of viral pathogens to infect humans. Furthermore, our focussed classification approach demonstrates the power of modern image-processing algorithms to break the shackles of symmetry that have limited our understanding of virus structural biology for so long.

## Results

**Fig 1:**
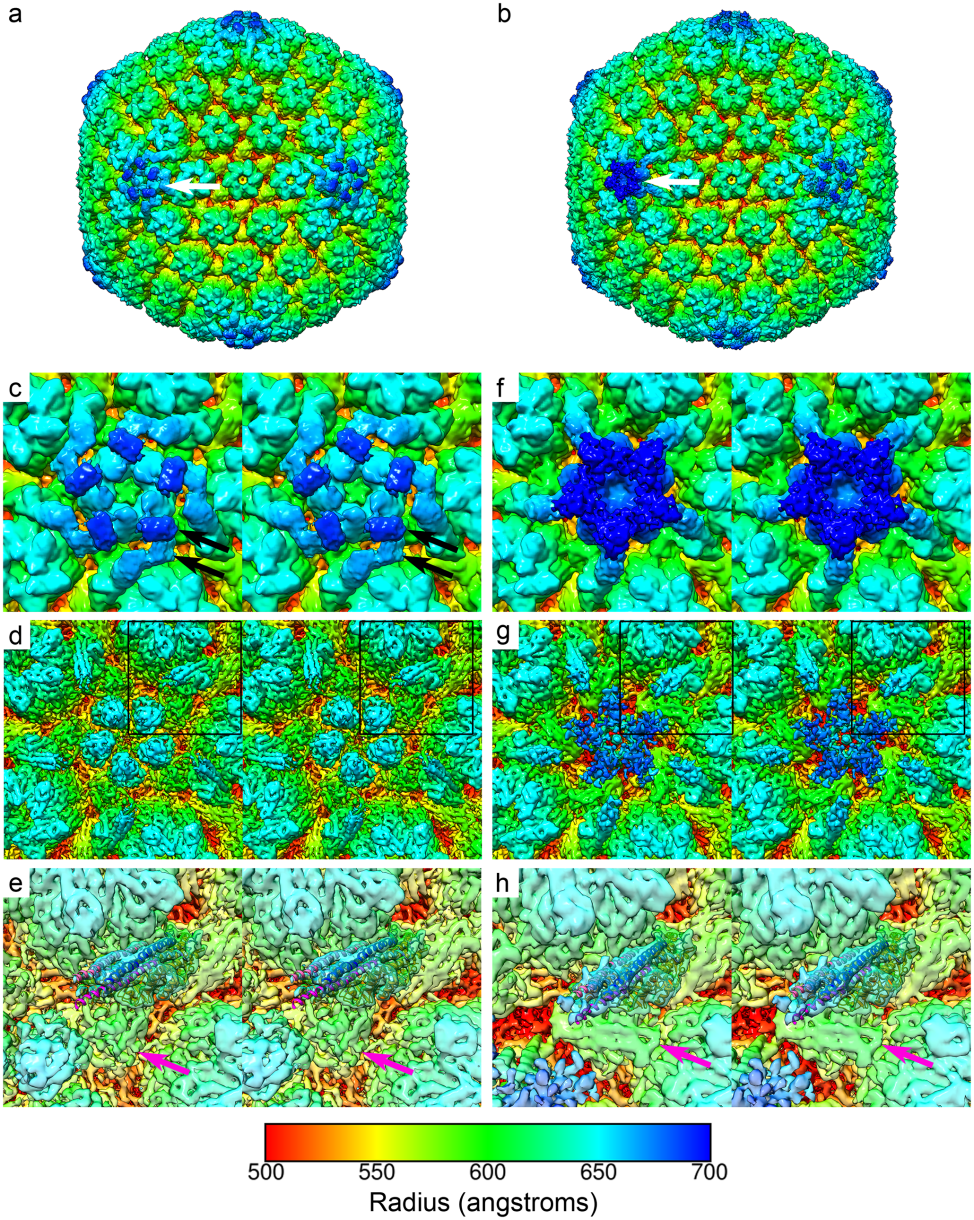
CryoEM and 3D image reconstruction of HSV-1 virions. Views of unsharpened 3D reconstructions and close-up stereo pair images of the penton and portal vertices. Imposition of full icosahedral symmetry led to the calculation of a map at 6.3 angstroms resolution (a). At each 5-fold symmetry axis, the CATC binds to peripentonal triplexes to form an assembly that lays over the major capsid protein penton (white arrow, a). A sharpened map reveals higher-resolution features and in particular the CATC five-helix bundle (d,e). Focussed classification led to the calculation of a C5 symmetric reconstruction at 7.7 angstroms resolution (b). This revealed the structure of the unique portal-vertex (white arrow, b). In the sharpened map, the CATC five-helix bundle is also well resolved (g,h). A close-up view of the penton-vertex highlights the structure of the CATC—in particular, the two globular densities that have been attributed to the C-terminal domain of pUL25 (black arrows, c). The CATC five-helix bundle is highlighted by fitting of atomic coordinates for this assembly (extracted from PDB 6CGR) (boxed region, d); pUL17 is shown as an orange ribbon; two copies of pUL25 (N-terminal domains) are shown in shades of blue; and two copies of pUL36 (C-terminal domains) are shown in shades of pink (e). The Ta triplex is indicated by a pink arrow (e). A close-up view of the portal-vertex shows that the 5-fold symmetry axis is capped by a tiered structure comprising two star-shaped rings of density (the PVAT, f). The arms of the CATC are also angled more towards the 5-fold axis. The sharpened map, viewed at a higher threshold, does not show the distal tier of the PVAT (g). Rigid body docking of CATC coordinates gave a good fit to the density and revealed a 6° counter-clockwise rotation of this assembly compared to that of the penton-vertex (compare e and h). Interestingly, we see that the position occupied by the Ta triplex in the penton-vertex is occupied by a much larger globular density at the portal-vertex (pink arrow). All maps are coloured according to radius in angstroms (see colour key). CATC, capsid-associated tegument complex; cryoEM, electron cryomicroscopy; HSV, Herpes Simplex Virus; PVAT, portal-vertex–associated tegument.

To determine the structure of the unique 5-fold vertex comprising the portal and portal-vertex–associated tegument (PVAT), we imaged purified HSV-1 virions by cryoEM (S1 Fig). A total of 3,702 micrographs were captured, from which a dataset of 6,069 virion images was extracted for 3D reconstruction. An initial 3D reconstruction was calculated with full icosahedral symmetry imposed in order to accurately define the particle origins and orientations in each image (Fig 1a). The icosahedral reconstruction achieved a resolution of 6.3 Å (S2 Fig, S1 Data) and closely resembled recently published structures at a similar resolution, revealing well-defined CATC density with a clear five-helix bundle that has been shown to comprise helices donated by a single copy of pUL17, two copies of pUL25, and two copies of pUL36 (Fig 1d and 1e) [5,32]. At lower isosurface threshold, we see two distinct globular domains per CATC, one on top of the pentonal pUL19 and one lying to the side. These densities have been shown to be the C-terminal domain of pUL25; thus, there are 10 pUL25 molecules per 5-fold vertex (Fig 1c) [5].

Following the refinement of our icosahedral reconstruction, focussed classification was performed to identify and reconstruct the unique portal-vertex. A 3D cylindrical mask was created to focus the 3D classification analysis onto a single 5-fold symmetry axis. A metadata file was created to contain particle orientations with expanded icosahedral symmetry such that each particle image had 60 orientations assigned, corresponding to the 60-fold redundancy of an icosahedral object. These data were subjected to 3D classification to calculate 10 3D reconstructions of the masked 5-fold vertex. A single class was identified that showed density significantly different from the known structures of the penton-vertex. Interrogation of the metadata for this class showed that the majority of particle images contributed five views (S3 Fig, S2 Data). This is consistent with there being a single portal structure per virion, and as a consequence, C5 symmetry is imposed on our reconstruction by the data.

### Structure of the PVAT

A relaxed symmetry (C5) reconstruction was calculated yielding a map at a resolution of 7.7 Å (Fig 1b, S4 Fig, S3 Data). This revealed a uniquely structured capsid-associated tegument assembly at the portal-vertex (Fig 1f–1h, S1 Movie), giving rise to the tail-like features previously described at lower resolution, termed the PVAT [35]. At intermediate resolution, we see that the portal-vertex CATC density closely resembles that seen at the penton-vertices. The five-helix bundle, comprising pUL17, pUL25, and pUL36 is well resolved but is rotated approximately 6° counter-clockwise relative to that of the penton-vertex. In penton-vertices, this part of the CATC is anchored to the capsid by binding to two triplexes, the peripentonal Ta triplex, and the Tc triplex. At the portal-vertex, the CATC still binds to the Tc triplex; however, the Ta triplex is replaced with a globular density that appears almost twice the size of a normal triplex (pink arrow, Fig 1e and 1h). At this resolution, it is unclear whether this feature is made up of a heterotrimer of pUL38 and pUL18, plus an additional component, or whether the triplex has been entirely substituted by another protein. A candidate for this additional protein density is pUL36. This gene product was recently shown to be a component of the CATC, contributing two copies of its C-terminal domain to the five-helix bundle; however, only a small proportion of that large protein has thus far been accounted for (47 of 3,164 amino acids) [5,21].

**Fig 2:**
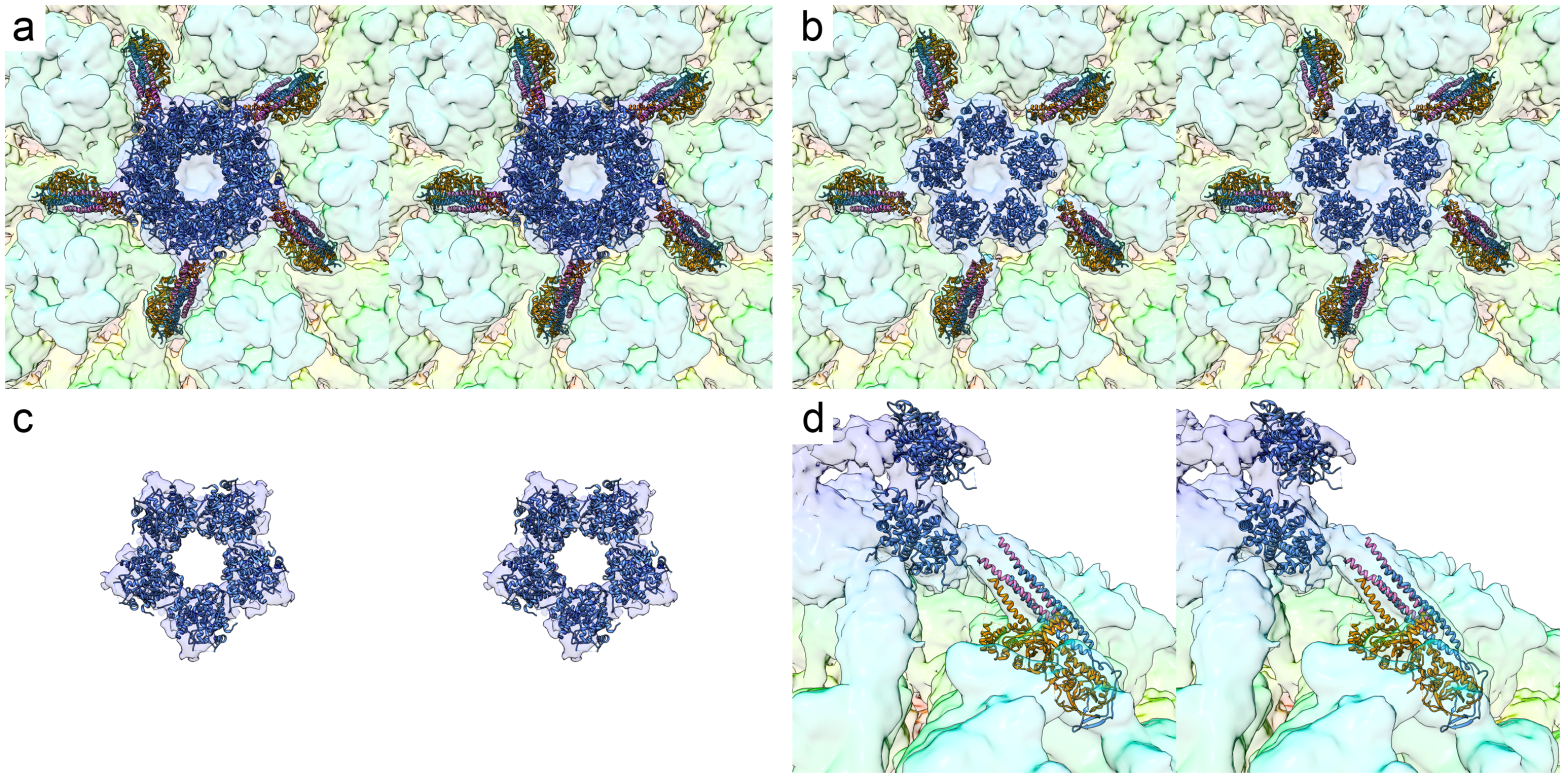
Stereo pair views of the structure and composition of the PVAT. Atomic coordinates for the CATC components pUL17, pUL25, and pUL36 (PDB 6CGR [5]) and the C-terminal domain of pUL25 (PDB 2F5U [38]) were docked into the portal-vertex density (a). Each pentonal five-helix CATC bundle has been shown to include two copies of an N-terminal α-helix of pUL25 (blue) along with two copies of a C-terminal α-helix of pUL36 (pink); these are bound to pUL17 (orange) [5]. The atomic model of the penton-vertex CATC matches well the equivalent density at the portal-vertex, indicating that there are likely a total of 10 copies of pUL25 at the portal-vertex as well. The PVAT assembly comprises 10 globular densities arranged as two C5 symmetric star-shaped rings that crown the portal-vertex. We docked five copies of the atomic model for the C-terminal domain of pUL25 into the proximal (inner) tier (blue, b). The docked coordinates were then saved as a single model that was docked into the less well-defined distal tier (c). A side view of the CATC/PVAT components is shown (d). CATC, capsid-associated tegument complex; PVAT, portal-vertex–associated tegument.

As well as the novel triplex-like density, we see major differences in the arrangement of the C-terminal domains of pUL25 at the portal-vertex. Peripentonal CATC complexes have two globular densities that bind to penton pUL19: one on top and one to the side of each copy of the major capsid protein (black arrows, Fig 1c). Portal-vertex CATC also shows 10 globular densities that we attribute to the pUL25 C-terminal domains. These are however arranged to form two star-shaped tiers to make up the tail-like PVAT. The distal (outermost) tier being rotated approximately 36° relative to the proximal one (Fig 2, S2 Movie). As was seen in penton vertices of our icosahedral reconstruction and published structures [5,21], the C-terminal domains of pUL25 are less well resolved than the CATC five-helix bundle; in particular, the distal tier is only visible at lower threshold levels and is more clearly seen in unsharpened maps, suggesting that this feature is not rigidly constrained. Sharpening is a process of weighting data across different resolution ranges to compensate for the loss of high-resolution information during imaging and data-processing—effectively, it down-weights low-resolution features to reveal finer details. Sharpened maps are typically shown at a higher isosurface threshold level, however density arising from flexible regions may only present low-resolution features and can disappear in sharpened maps.

Atomic coordinates for the CATC five-helix bundle (extracted from PDB 6CGR [5]) and the C-terminal domain of pUL25 (PDB 2F5U [38]) were docked to the PVAT density. The CATC bundle fitted the density well, suggesting that the assembly, although slightly displaced, is not radically different from that at penton vertices (Fig 1h). Given the limited resolution in the density that we attribute to the pUL25 C-terminal domains, however, docking of these coordinates is only shown to illustrate our interpretation; there is insufficient detail to produce a reliable fit (Fig 2, S2 Movie).

### Structure of the portal-vertex interior

**Fig 3:**
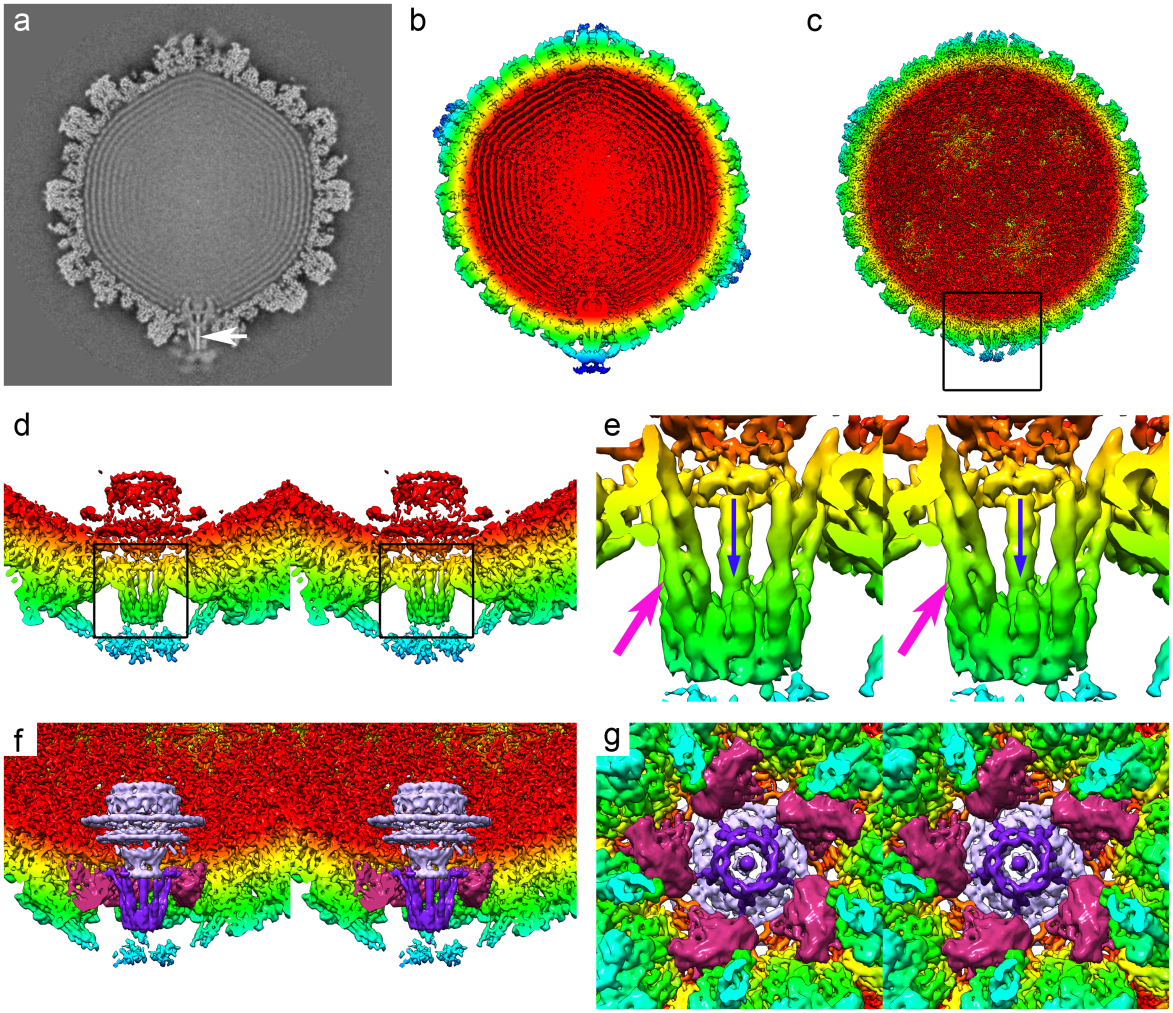
The structure of the portal-vertex interior. A central slice through the C5 reconstruction of the HSV-1 virion reveals the internal features of the portal-vertex (a). Notably, a strong linear density is seen to run through the portal-vertex that we attribute to genomic DNA (white arrow). The outermost feature, the PVAT, is weakly resolved as fuzzy density, suggesting that this feature is not well constrained. Isosurface representation of the unsharpened map presents a clearer representation of the PVAT (b), while in the sharpened density map, the packaged DNA is not seen, revealing the interior features of the capsid shell (c). A clipped, close-up view of the portal-vertex (boxed in c) highlights the morphology of the portal (pUL6) and, lying between the portal and the PVAT, the pentameric portal-vertex protein (wall-eyed stereo pair view, d). A close-up stereo view of the pentameric portal-vertex protein (boxed in d) clearly shows the density that is consistent with a two-helix coiled-coil motif (pink arrow, e). The density running through the centre of the portal-vertex that we attribute to DNA is also clearly visible (blue arrow). The density map was segmented to highlight three features: the portal (mauve), the pentameric portal-vertex protein (purple), and the periportal triplex–like density (magenta). The segmented portal-vertex is presented as stereo views both perpendicular to (e) and along (f) the portal axis. In panel e, the capsid and triplex-like assemblies are clipped to expose the pentameric portal-vertex protein; this and the portal are not clipped. In panel f, the pUL25/PVAT component is clipped away to expose the underlying features. HSV, Herpes Simplex Virus; PVAT, portal-vertex–associated tegument.

A central slice through the 5-fold symmetrical reconstruction of the herpes simplex virion reveals the internal features of the portal-vertex and the packaged DNA (Fig 3a, S3 Movie). Lying just inside the capsid shell, we can see poorly defined density that we attribute to the portal protein pUL6 (Fig 3c and 3d). Single-particle 3D reconstruction of this assembly has previously shown it to have cyclic symmetry, forming oligomers ranging from undecamers to tetradecamers. Authentic pUL6 portals assemble as dodecamers [39]. Thus, a symmetry mismatch between this structure and the C5 capsid leads to incoherent averaging and explains the lack of high-resolution features seen in our reconstruction. Nonetheless, we can segment this feature from our density map to highlight its position and gross morphology (Fig 3f and 3g). Running through the centre of the portal-vertex along the 5-fold axis, we see strong density that extends through the portal and up against the inner surface of the PVAT. This is highlighted with a white arrow in Fig 3a and in S3 Movie. We interpret this as being the trailing end of the viral DNA, retained in the portal-vertex and ready to be ejected upon infection of a new host cell. A similar feature has been shown in the bacteriophages Ø29 and Spp1, in which specific interactions between stopper proteins and the DNA hold the end of the DNA within the tail assembly [40,41].

Lying between the portal and the pUL25 PVAT density, a novel 5-fold symmetrical assembly replaces the usual pUL19 penton. This structure is composed of five protein subunits that are largely α-helical. In each subunit, a well-resolved two-helix coiled coil extends radially and is approximately 10 nm in length (Fig 3e). This structure is anchored to the capsid through an interaction with the Ta triplex–like structures that are arranged about the portal-vertex. At this resolution, we see no evidence of an interaction with the major capsid protein. The tapered end of the portal inserts into this assembly; however, owing to the symmetry mismatch, we are unable to identify specific contacts between the two structures. The identity of the pentameric portal-vertex protein is unclear. However, as the locations of all the components of the icosahedral capsid shell and the pUL6 portal protein are known, together with the CATC proteins, pUL17, and most of pUL25, a candidate for this density is also the inner tegument protein, pUL36. As noted above, pUL36 is known to be a CATC component; however, this represents only a small proportion of this large protein, leaving the rest unaccounted for.

Another possible candidate for this novel assembly is the terminase protein pUL33. In tailed phages, terminase complexes usually comprise two proteins: a small subunit that mediates binding of the terminase to the viral DNA and a large subunit (having both endonuclease and ATPase activity) that powers the translocation of the genome into the capsid through the portal and then cleaves the DNA when the head is full or a cleavage signal is detected. The HSV-1 terminase is made up of three proteins: pUL15, pUL28, and pUL33 [16]. pUL28 is functionally equivalent to the small terminase subunit [19], and pUL15 shows sequence similarity to the large terminase subunit of phage. The function of pUL33 is rather less well defined. It is predicted to be largely α-helical in structure [42], and thus we do not rule out the possibility that the novel pentameric portal-vertex protein could be pUL33. By analogy to bacteriophage terminase complexes, however, the terminase assembly is expected to detach from the portal following cleavage of the concatamer, remaining associated with the unpackaged DNA, ready to engage the portal in another empty procapsid. Furthermore, proteomics analysis has failed to detect terminase components in purified HSV-1 virions [43]. Further biochemical and structural analysis of isolated nucleocapsids and virions is therefore required to unambiguously identify this protein.

#### Herpesviruses package their DNA genomes as a left-handed spool

**Fig 4:**
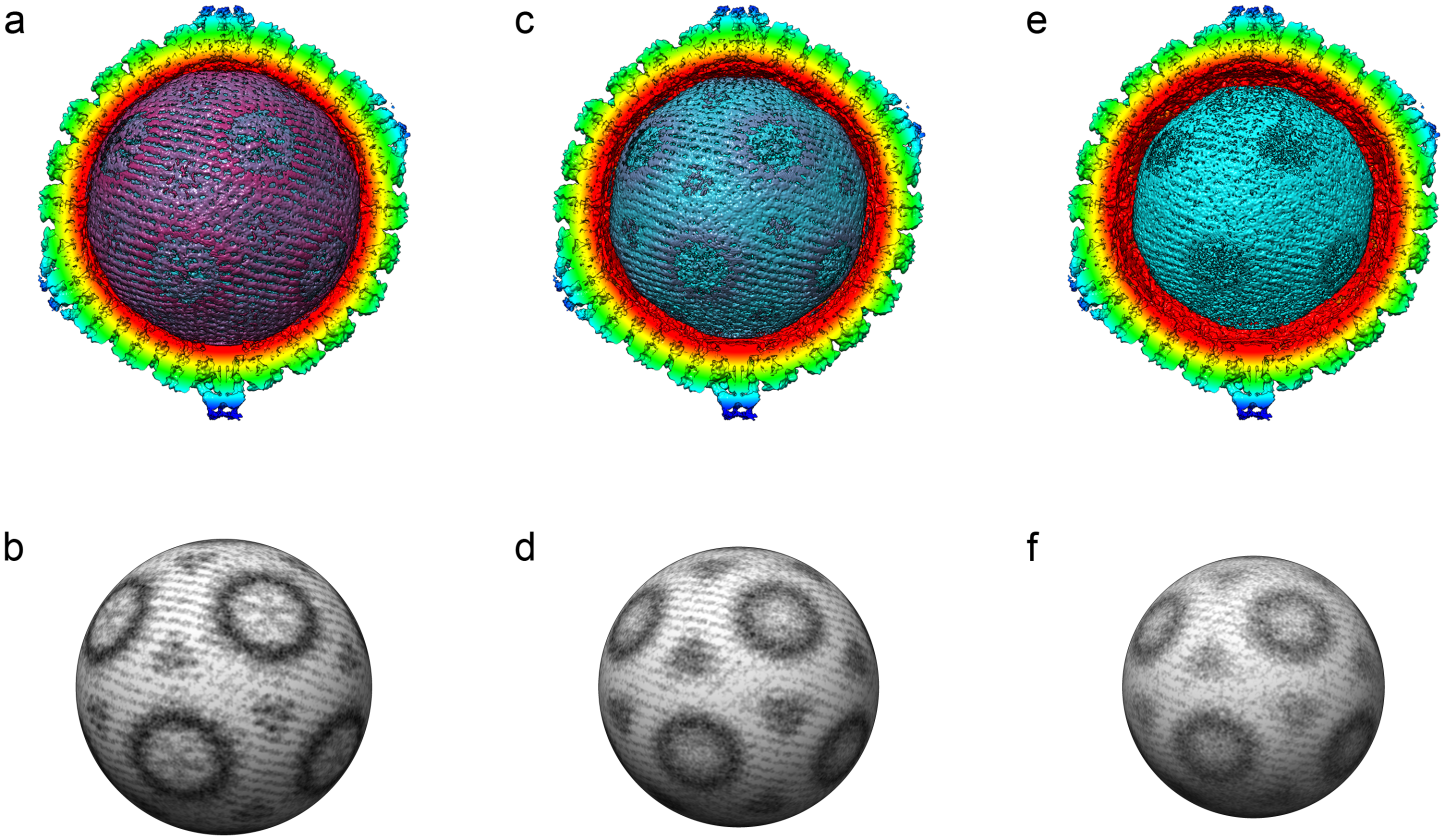
DNA packaging in HSV-1. The unsharpened C5 reconstruction of HSV-1 is presented showing a series of radially cropped views of the interior density; these reveal the highly ordered arrangement of packaged DNA. The outermost (a), second (c), and third (e) shells are shown, revealing a left-handed spool of density. Spherical sections of each shell are also presented (b,d,f). HSV, Herpes Simplex Virus.

In addition to the much-improved view of the HSV-1 portal-vertex, our data also reveal detailed structural information for the packaged DNA. Our low-symmetry reconstruction reveals that the DNA is packed as a left-handed spool—that is, the strands of DNA are seen to rotate clockwise as they ascend when viewed perpendicular to the portal axis (Fig 4, S4 Movie). The outermost three layers of DNA clearly show an identical orientation of the spooled DNA. The presence of clear density for the genomic DNA indicates that a very consistent packaging process occurs when DNA is pumped into the nascent nucleocapsid. Our data suggest that the bending forces on the incoming DNA, combined with lateral or rotational forces imparted by the portal motor, lead to a consistent orientation for the DNA spool inside every virion. Herpesvirus DNA is packed to a very high density [44] (indeed, in another herpesvirus, cytomegalovirus, genome density approaches the limits of what may occur without transition to a crystalline state [45]). It is known that both pUL25 and pUL36 are critical to the retention of packaged DNA. Our data suggest a reason for this, showing that pUL25 forms a double-layered cap on the outer face of the portal-vertex (the PVAT). We can see strong density that we interpret as being DNA running through the central channel of the portal-vertex, extending through both the pUL6 portal and the unidentified pentameric portal-vertex protein complex, reaching to the distal tip of the latter structure and terminating at the position occupied by the PVAT/pUL25 density.

### Summary

We have applied a novel image-processing approach to determining the structure of an important asymmetric feature of the icosahedral herpesvirus nucleocapsid. Our data provide new insights into the organisation and composition of this important molecular machine. The herpesvirus portal-vertex is the unique site through which the viral genome both enters and exits the nucleocapsid. It is therefore a critical assembly in both virion morphogenesis and initiation of infection. We show that the PVAT, a tail-like structure previously described at low resolution, is likely composed of the C-terminal domain of pUL25, a protein known to be critical to DNA retention. We have also described two novel structures. The first, a hitherto unknown pentameric assembly, lays between the portal and PVAT in place of the pUL19 penton and has a clear two-helix coiled-coil structure. The second is a large assembly that occupies the space usually taken up by the Ta triplex in peripentonal vertices. We have suggested possible identities for both structures; however, further biochemical analysis, coupled with higher-resolution structure determination, are required to provide a definitive description. In agreement with previous lower-resolution studies that employed electron tomography, we show that the portal motor, composed of 12 copies of pUL6, lays against the inner edge of the capsid shell. Finally, our data show that HSV-1 genomic DNA is packaged as a left-handed spool arranged in concentric layers. This suggests that the process of genome translocation combined with interactions between the inner surface of the capsid and successive layers of DNA lead to a highly reproducible packing process. This is perhaps not as remarkable as one might initially surmise. Herpesviruses package their genomes to extremely high density. It is critical, then, that the genome should be able to reliably achieve this high density and, with equal reliability, eject the complete genome through the nuclear pore to initiate infection.

## Materials and methods

### Culture and purification of HSV-1 virions

Herpes simplex virus was propagated in BHK cells grown in roller bottles containing growth medium (GMEM, 10% FCS, 10% tryptose phosphate broth). Confluent cells were infected with HSV-1 strain 17syn+ at a m.o.i. of 0.002 pfu/cell. Cells were incubated at 37 °C for 3 days, then harvested by shaking into the medium. Cells and media were centrifuged at 1600× g for 10 minutes to remove cell debris. The supernatant was then transferred to a new tube and spun at 17,000× g for 2 hours. The pellet was then gently resuspended on ice overnight by overlaying with 2 ml GMEM. The resuspended material was moved to a new tube and clarified by spinning at 200× g for 10 minutes. The supernatant was layered onto the top of a 5%–15% Ficoll gradient prepared in GMEM and centrifuged at 26,000× g for 2 hours. The opaque band containing the virions was collected by side puncture of the tube using an 18-gauge needle. Collected virions were diluted in GMEM and pelleted at 40,000× g for 1 hour. The pellet was washed gently in PBS and then allowed to resuspend in 50–100 μl PBS by incubating on ice for at least 1 hour.

### CryoEM

HSV virions were prepared for cryoEM by plunge freezing into liquid ethane. Four μl of purified virions was loaded onto a freshly glow-discharged holey-carbon support film (R2/2 Quantifoil) in an FEI Vitrobot mk. IV vitrification robot. The grid was immediately blotted for 3 seconds and then plunged into liquid nitrogen–cooled liquid ethane. Grids were imaged at the United Kingdom national cryoEM facility (electron bioimaging centre—eBIC) at Diamond Light Source, Harwell, in an FEI Titan Krios cryotransmission electron microscope at a nominal magnification of 81,000×. Images were recorded as ‘movies’ on a Falcon III camera operated in integrating mode with sampling of 1.78 Å/pixel. Movies were recorded as 12-second exposures, giving a total of 480 detector frames that were integrated into 40 fractions, with a dose rate of 1.95 e/Å^2^/fraction.

### Image processing

All image processing was performed using Relion 2.1 [46] on a GPU workstation running Linux CentOS 7. 3,702 micrograph movies were processed to correct for particle movement using Motioncor2 [47], and the defocus for each motion-corrected micrograph was estimated using GCTF [48]. A small subset of particles was picked and subjected to 2D classification to produce a template for automated particle picking, which yielded a total of 12,431 virion images for further processing. Particles were initially extracted with 2× binning. After 2D classification of this dataset 7,476 particles were selected for ab initio calculation of a starting model, followed by 3D classification (imposing full icosahedral symmetry in both cases). This led to the definition of a final dataset of 6,069 virion images that were taken forward for 3D refinement with full icosahedral symmetry. Once completed, a new dataset of particles with 1.5× binning was extracted and used to calculate the final icosahedral reconstruction.

Following icosahedral reconstruction, focused classification was performed to identify the unique portal-vertices [36,37]. A cylindrical mask was prepared in SPIDER [49] to cover a single 5-fold symmetry axis. A metadata file (STAR file) was generated to expand the symmetry for our dataset, i.e., for each virion image, 60 orientations were defined, corresponding to the 60 symmetry-related views of the icosahedral object. To speed up the calculation, a new dataset was extracted from the raw micrographs with 5× binning. These data were then subjected to masked 3D classification, with a T value of 20, to reconstruct a single 5-fold vertex and classify the data into self-similar classes. During this process, orientations and origins were not refined. A total of 10 classes were calculated, one of which was identified as containing the unique portal-vertex. To calculate our final C5 symmetric reconstruction, we used 1.5×-binned particle images.

Resolution assessment was performed in Relion, using the postprocessing task to mask the density maps and calculate the ‘gold-standard’ Fourier shell correlation. A B-factor was estimated and applied to each reconstruction [50], which was then interpreted by visualization in UCSF Chimera [51]. Docking of atomic coordinates was performed using the ‘fit model in map’ function of UCSF Chimera. Segmentation was performed using the ‘Segger’ plugin in UCSF Chimera.

The C5 reconstruction has been deposited in the EM databank with accession code EMD-4347. Motion-corrected micrographs (raw data) are deposited in EMPIAR with accession code EMPIAR-10189.

## Supporting information

S1 Fig Cryomicrograph of HSV-1 virions. Scale bar = 100 nm. HSV, Herpes Simplex Virus.
(TIF)

S2 Fig Fourier shell correlation plot for the icosahedral reconstruction of HSV-1. HSV, Herpes Simplex Virus.
(TIF)

S3 Fig Focussed classification to identify the portal-vertex in HSV-1. To solve the structure of the portal-vertex, focussed classification was used. This allows us to determine the structures of asymmetric features in high-symmetry objects. To achieve this, we expand the symmetry of the dataset such that each particle image has multiple orientations specified according to the redundancy of the symmetry group. In the case of icosahedral objects, each particle would contribute 60 views. A metadata file was therefore created in which each particle image was assigned 60 symmetry-related orientations based on the single orientation that had been determined during 3D reconstruction with full icosahedral symmetry. Masked 3D classification, focussing on a single 5-fold axis, led to the definition of a class that showed density significantly different from the known penton-vertex structure. To understand the distribution of particle views present in this class, we sought to determine the number of times each particle was assigned to it. From our dataset of 6,069 virion images, we produced a metadata file containing 364,140 putative views (60 × 6,069). The portal-vertex class was found to contain 26,891 entries (approximately 7.4% of the total dataset). There are twelve 5-fold symmetry axes in an icosahedral object; thus, we would expect 8.3% (1/12) of the data to be assigned to the unique portal-vertex. Further interrogation of the metadata file for this class revealed that 5,337 unique particles were represented in the dataset; thus, 732 particles did not present portal density that was readily identified by our analysis. We determined the number of views for each virion image in the portal-vertex class; this revealed that the median number of views per particle was 5, consistent with the C5 symmetry of the portal axis and indicating that most (if not all) particles have only one portal. HSV, Herpes Simplex Virus.
(TIF)

S4 Fig Fourier shell correlation plot for the C5 reconstruction of HSV-1. HSV, Herpes Simplex Virus.
(TIF)

S1 Data Fourier shell correlation data for the icosahedral reconstruction of HSV-1. Data used to generate S2 Fig. HSV, Herpes Simplex Virus.
(XLSX)

S2 Data Coordinates for each particle/view that contributed to the C5 symmetric reconstruction of HSV-1. Data used to generate S3 Fig. These data include particle coordinates and micrograph names that should allow readers to extract the particles from raw micrographs deposited in the EMPIAR databank—EMPIAR 10189. HSV, Herpes Simplex Virus.
(XLSX)

S3 Data Fourier shell correlation data for the C5 reconstruction of HSV-1. Data used to generate S4 Fig. HSV, Herpes Simplex Virus.
(XLSX)

S1 Movie Movie to show the structure of CATC proteins at the portal and penton vertices. CATC, capsid-associated tegument complex.
(MOV)

S2 Movie Movie to show the structure of the portal-vertex associated tegument protein. The atomic coordinates for the C-terminal domain of pUL25 (PDB 2F5U) were docked to the PVAT density. Five copies were docked to the proximal tier of the PVAT; the fitted coordinates for all five molecules were saved as a single model that was then docked to the less well-defined distal tier density. Coordinates for the CATC five-helix bundle were also docked (PDB 6CGR). CATC, capsid-associated tegument complex; PVAT, portal-vertex–associated tegument.
(MOV)

S3 Movie Movie to show the structure of internal features at the portal-vertex. Sections through the 3D reconstruction are presented to show the internal density of the portal-vertex. Isosurface representations are shown annotated to highlight the structure of the pentameric portal-vertex protein, the portal (pUL6), and DNA running through the centre of the portal-vertex.
(MOV)

S4 Movie Movie to show the arrangement of DNA within the HSV capsid. HSV, Herpes Simplex Virus.
(MOV)

## Abbreviations

CATC: capsid-associated tegument complex
cryoEM: electron cryomicroscopy
HSV: Herpes Simplex Virus
PVAT: portal-vertex–associated tegument
