# Supplementary figures and images for: Structure of the herpes simplex virus portal-vertex
Source: PLoS Biol. 2018 Jun 20;16(6):e2006191. doi: 10.1371/journal.pbio.2006191 (PMC6028144; doi:10.1371/journal.pbio.2006191)

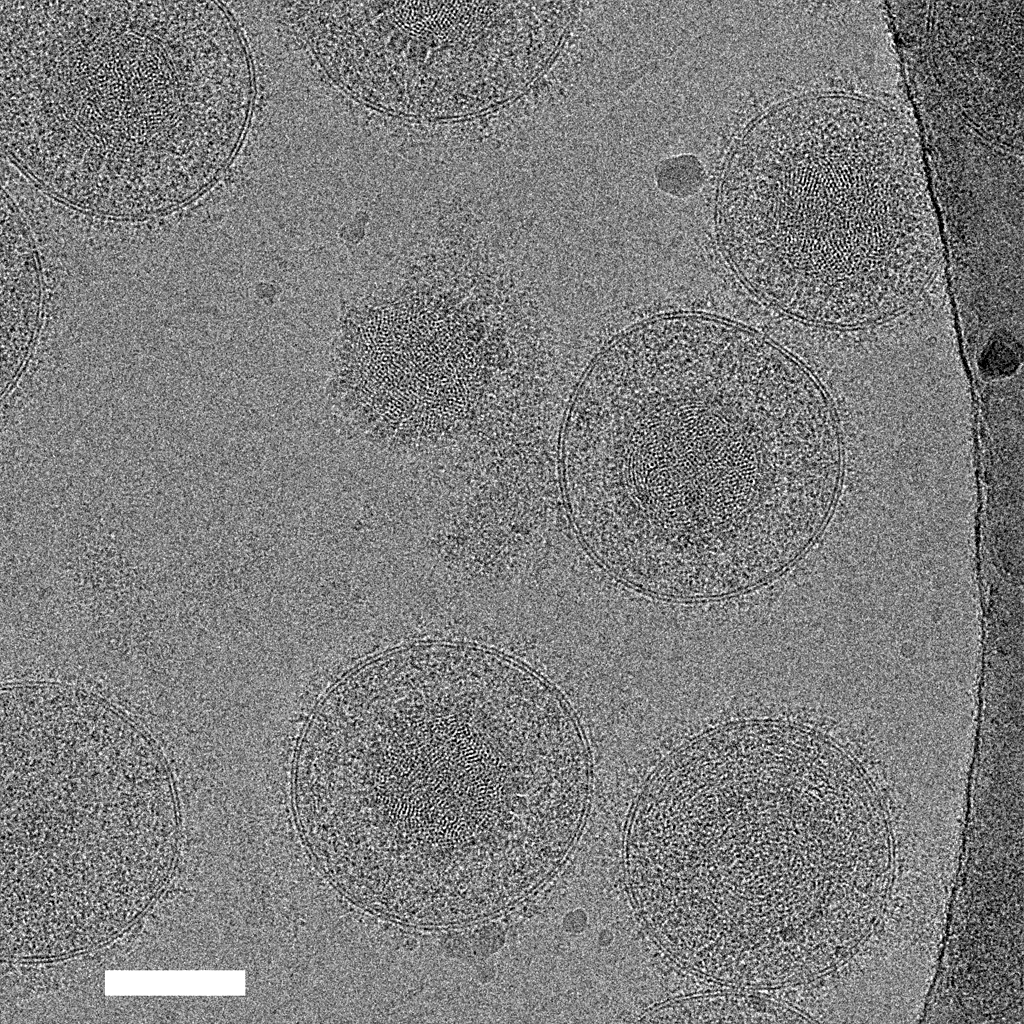

Supplement: S1 Fig — Scale bar = 100 nm. HSV, Herpes Simplex Virus. (TIF) [file pbio.2006191.s001.tif]

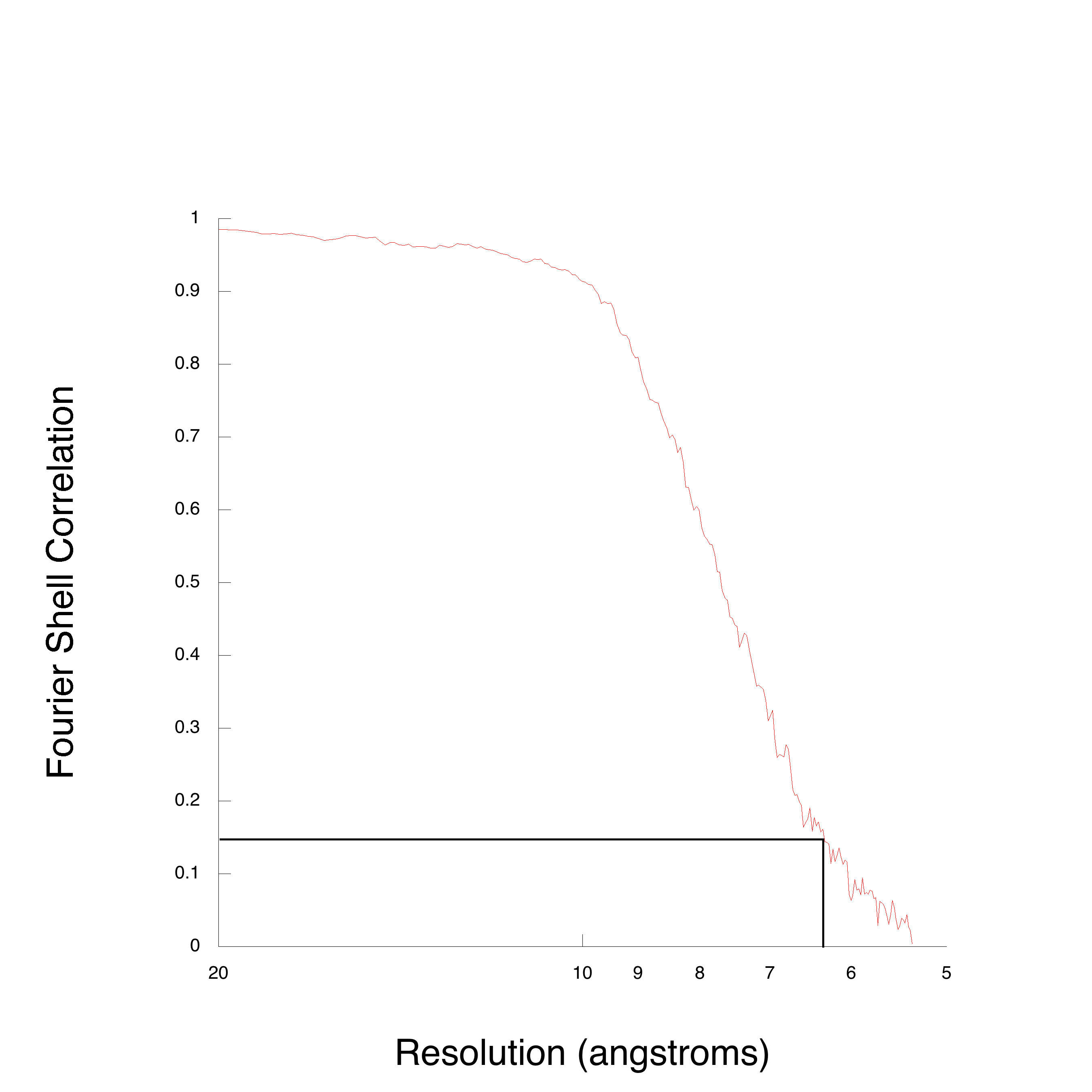

Supplement: S2 Fig — HSV, Herpes Simplex Virus. (TIF) [file pbio.2006191.s002.tif]

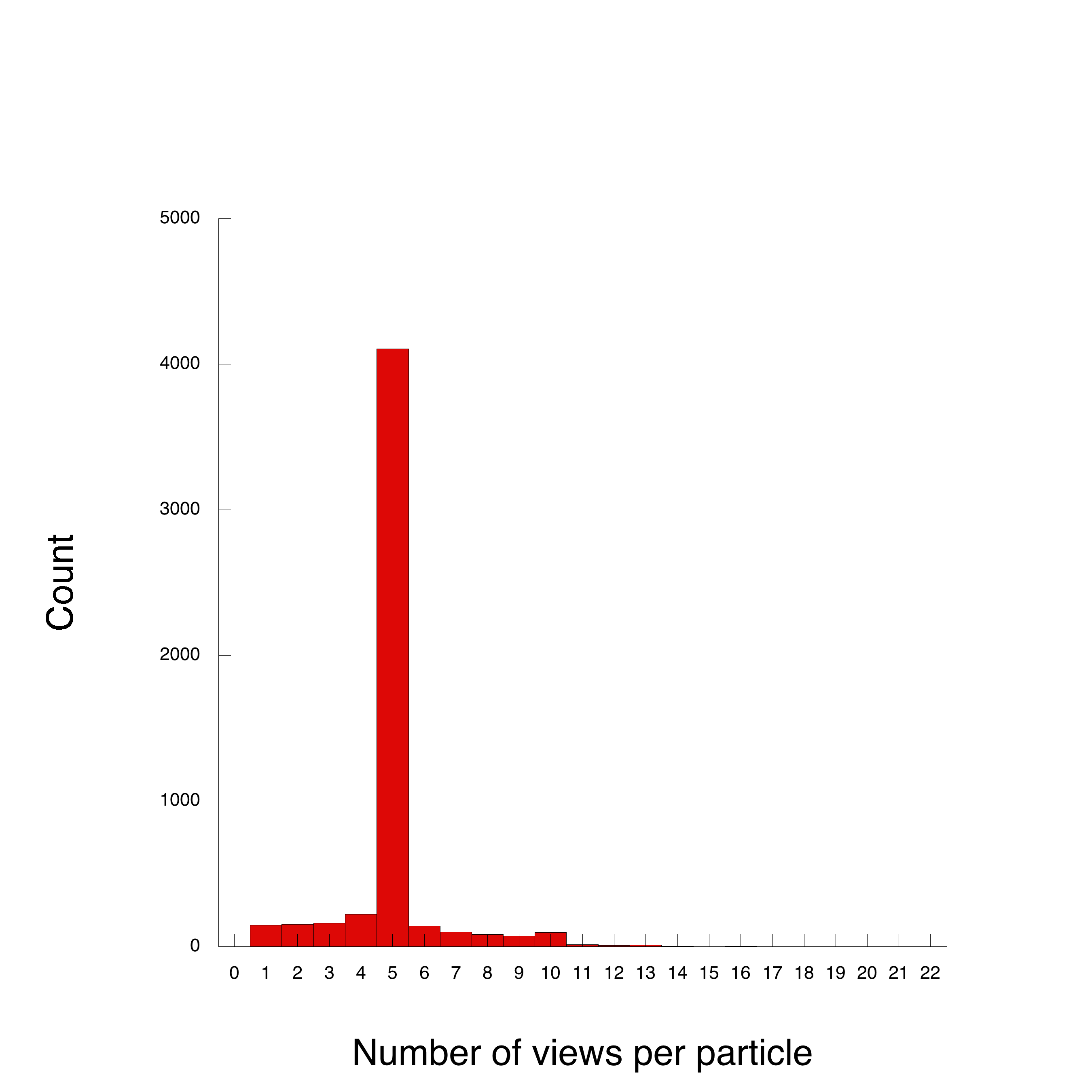

Supplement: S3 Fig — To solve the structure of the portal-vertex, focussed classification was used. This allows us to determine the structures of asymmetric features in high-symmetry objects. To achieve this, we expand the symmetry of the dataset such that each particle image has multiple orientations specified according to the redundancy of the symmetry group. In the case of icosahedral objects, each particle would contribute 60 views. A metadata file was therefore created in which each particle image was assigned 60 symmetry-related orientations based on the single orientation that had been determined during 3D reconstruction with full icosahedral symmetry. Masked 3D classification, focussing on a single 5-fold axis, led to the definition of a class that showed density significantly different from the known penton-vertex structure. To understand the distribution of particle views present in this class, we sought to determine the number of times each particle was assigned to it. From our dataset of 6,069 virion images, we produced a metadata file containing 364,140 putative views (60 × 6,069). The portal-vertex class was found to contain 26,891 entries (approximately 7.4% of the total dataset). There are twelve 5-fold symmetry axes in an icosahedral object; thus, we would expect 8.3% (1/12) of the data to be assigned to the unique portal-vertex. Further interrogation of the metadata file for this class revealed that 5,337 unique particles were represented in the dataset; thus, 732 particles did not present portal density that was readily identified by our analysis. We determined the number of views for each virion image in the portal-vertex class; this revealed that the median number of views per particle was 5, consistent with the C5 symmetry of the portal axis and indicating that most (if not all) particles have only one portal. HSV, Herpes Simplex Virus. (TIF) [file pbio.2006191.s003.tif]

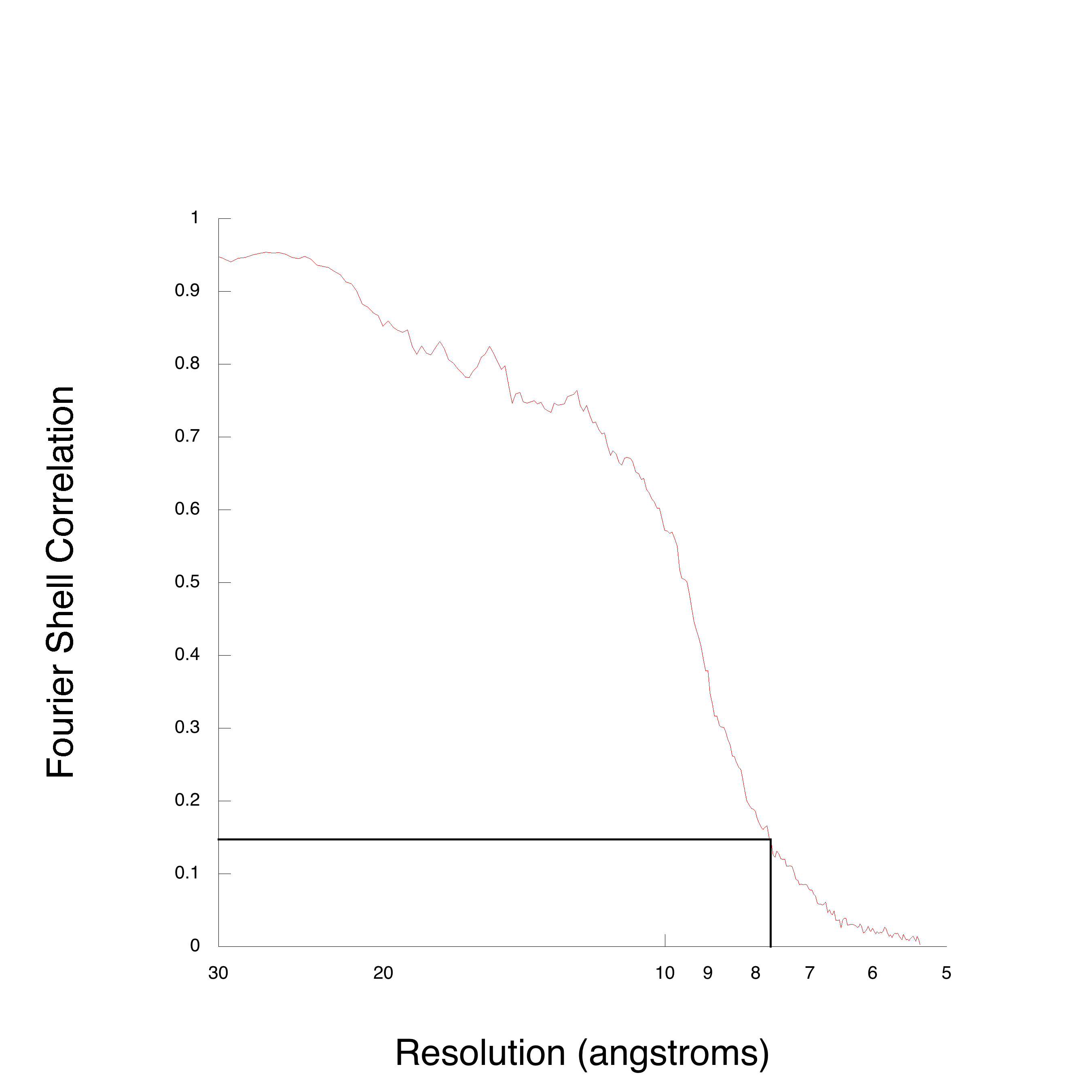

Supplement: S4 Fig — HSV, Herpes Simplex Virus. (TIF) [file pbio.2006191.s004.tif]
